# Differences in the Occurrence of Cell Wall Components between Distinct Cell Types in Glands of *Drosophyllum lusitanicum*

**DOI:** 10.3390/ijms242015045

**Published:** 2023-10-10

**Authors:** Bartosz J. Płachno, Małgorzata Kapusta, Piotr Stolarczyk, Piotr Świątek, Irene Lichtscheidl

**Affiliations:** 1 Department of Plant Cytology and Embryology, Institute of Botany, Faculty of Biology, Jagiellonian University in Kraków, 9 Gronostajowa St., 30-387 Kraków, Poland; 2 Laboratory of Electron Microscopy, Faculty of Biology, University of Gdańsk, 59 Wita Stwosza St., 80-308 Gdańsk, Poland; malgorzata.kapusta@ug.edu.pl; 3 Department of Botany, Physiology and Plant Protection, Faculty of Biotechnology and Horticulture, University of Agriculture in Kraków, 29 Listopada 54 Ave., 31-425 Kraków, Poland; piotr.stolarczyk@urk.edu.pl; 4 Institute of Biology, Biotechnology and Environmental Protection, Faculty of Natural Sciences, University of Silesia in Katowice, 9 Bankowa St., 40-007 Katowice, Poland; piotr.swiatek@us.edu.pl; 5 Cell Imaging and Ultrastructure Research, University of Vienna, Althanstrasse 14, A-1090 Vienna, Austria; irene.lichtscheidl@univie.ac.at

**Keywords:** arabinogalactan proteins, carbohydrate epitopes, carnivorous plants, cell wall, Drosophyllaceae, digestive glands, mucilage glands, transfer cells, wall ingrowths

## Abstract

Carnivorous plants are mixotrophs that have developed the ability to lure, trap, and digest small organisms and utilize components of the digested bodies. Leaves of *Drosophyllum lusitanicum* have two kinds of glands (emergences): stalked mucilage glands and sessile digestive glands. The stalked mucilage glands perform the primary role in prey lure and trapping. Apart from their role in carnivory, they absorb water condensed from oceanic fog; thus, plants can survive in arid conditions. To better understand the function of carnivorous plant emergences, the molecular composition of their cell walls was investigated using immunocytochemical methods. In this research, *Drosophyllum lusitanicum* was used as a study system to determine whether cell wall immunocytochemistry differs between the mucilage and digestive glands of other carnivorous plant species. Light and electron microscopy were used to observe gland structure. Fluorescence microscopy revealed the localization of carbohydrate epitopes associated with the major cell wall polysaccharides and glycoproteins. The mucilage gland (emergence) consists of a glandular head, a connecting neck zone, and stalk. The gland head is formed by an outer and inner layer of glandular (secretory) cells and supported by a layer of endodermoid (barrier) cells. The endodermoid cells have contact with a core of spongy tracheids with spiral-shaped thickenings. Lateral tracheids are surrounded by epidermal and parenchymal neck cells. Different patterns of cell wall components were found in the various cell types of the glands. Cell walls of glandular cells generally are poor in both low and highly esterified homogalacturonans (HGs) but enriched with hemicelluloses. Cell walls of inner glandular cells are especially rich in arabinogalactan proteins (AGPs). The cell wall ingrowths in glandular cells are significantly enriched with hemicelluloses and AGPs. In the case of cell wall components, the glandular cells of *Drosophyllum lusitanicum* mucilage glands are similar to the glandular cells of the digestive glands of *Aldrovanda vesiculosa* and *Dionaea muscipula*.

## 1. Introduction

Carnivorous plants, in the course of evolution, have developed the ability to lure, trap, and digest small organisms, and make use of components from the digested bodies [1,2,3]. Charles Darwin was fascinated by these plants [4,5,6], which he called insectivorous [7], and even preferred research on this group of plants over others: “…*at this present moment I care more about* Drosera *than the origin of all the species in the world.*” (part of a letter from Charles Darwin to Charles Lyell) [8]. Interest in this ecological group of plants has been growing since Darwin’s time. Carnivorous plants are used as models for cytological, genomic, and physiological studies, e.g., [9,10,11,12]. 

During the evolution of the carnivory syndrome, carnivorous plants have used and modified many physiological processes that are also present in non-carnivorous ancestors. An example is jasmonate signaling, which in typical plants is involved in plant defense mechanisms, while in carnivorous plants it is used in recognition of captured prey and initiating the secretion of enzymes to digest prey, e.g., [13,14,15]. The repurposing of defense-related genes is an especially important trend in the evolution of plant carnivory [10,16,17].

The simple genetic changes in carnivorous plant genomes caused morphological evolution from simple leaves to sophisticated traps [17,18,19]. They are supplied with an appropriate glandular apparatus that can lure [20,21,22,23,24] and capture prey, e.g., fly-paper (adhesive) traps [25] and the pitchers of some *Nepenthes* with viscous pitcher fluid [26,27]. Nutrients from captured prey are absorbed by these digestive and absorptive organs [1,20,28,29] analogous to the stomach and intestine of animals [30]. The glandular apparatus of carnivorous plant traps (in Nepenthales and Lentibulariaceae) contains transfer cells [1,20,31,32]. These transfer cells participate in intensive short-distance transport between the symplast and apoplast [33,34]; thus, they may play an essential role in glands of carnivorous plants. 

**Figure 1:**
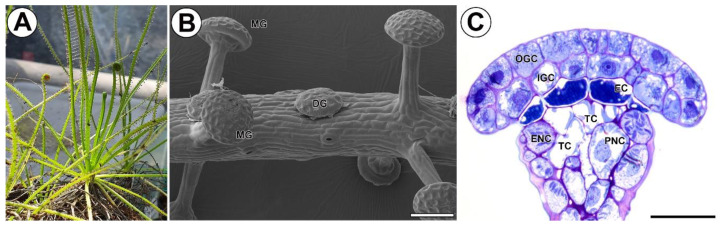
Distribution, morphology, and structure of mucilage glands of *Drosophyllum lusitanicum*. (**A**) *Drosophyllum lusitanicum* plants. (**B**) Part of a *Drosophyllum lusitanicum* leaf; stalked mucilage gland (MG), sessile digestive gland (DG), scale bar 100 µm. (**C**) Structure of head cells of the mucilage gland; outer glandular cell (OGC), inner glandular cell (IGC), endodermoid cells (EC), tracheid (TC), epidermal neck cell (ENC), parenchymal neck cells (PNC), scale bar 300 µm.

The order Nepenthales (containing five families) includes carnivorous plants that rely on three main methods of prey capture: pitcher traps (*Nepenthes*), snap traps (*Aldrovanda* and *Dionaea*), and fly-paper (adhesive) traps (passive traps: *Triphyophyllum* and *Drosophyllum*; active traps: *Drosera*) [35]. *Drosophyllum lusitanicum* (L.) Link (Drosophyllaceae) is a carnivorous subshrub up to 45 cm high with reverse circinate, linear leaves (Figure 1A). It is endemic to the western Iberian Peninsula and northern Morocco, where it grows in fire-prone Mediterranean heathlands on acid, nutrient-poor, dry soils [36,37,38,39]. The leaves of *Drosophyllum* have two kinds of glands (emergences): stalked glands producing a sticky mucilage, and sessile glands releasing digestive enzymes (Figure 1B). The morphology of these glands has been studied and described in detail by Fenner [40], Lloyd [36], and later in terms of ultrastructure by Schnepf [41,42]. 

The structure of the stalked mucilage glands resembles the stalked glands of *Triphyophyllum peltatum*, and—to some extent—also the emergences of the various *Drosera* species [43]. They serve to attract prey by volatile organic compounds in their mucilage [24] with “honey-like” odor [44], and the sticky mucilage glues the trapped animals to the leaf surface. Another important function, discovered only recently, is the provision of water to the plants; during the night, water from oceanic fog is condensed as dew onto the mucilage droplets and later absorbed partly into the leaves. This mechanism allows *Drosophyllum* to withstand the hot and arid conditions of its growth environment [45].

Using morphometry, Vassilyev [46] revealed that secretion of acid hydrolase in unstimulated digestive glands is continuous from the immature to mature state. He suggested that the subcellular mechanisms of hydrolase synthesis and secretion in carnivorous plant glands (in *Drosophyllum* as well as in the various species of Droseraceae) are analogous to the two subcellular strategies characteristic of the animal pancreas. Concerning uptake of extracellular substances, endocytosis was observed in the glands of *D. lusitanicum* [28,29]. Interestingly, endocytosis occurs not only in digestive glands assumed to be responsible for nutrition, but also in mucilage glands. 

For the understanding of both secretion and uptake of substance in glandular cells, the molecular composition of the cell walls surrounding the cytoplasm must be considered [47,48,49]. Based on our observations in previous studies of carnivorous plants *(Aldrovanda vesiculosa* and *Dionaea muscipula* [50,51]), we hypothesize that the cell walls of glandular cells should be rich in arabinogalactans and xyloglucans, especially since endocytosis and endocytic membrane networks are important for cell wall assembly and remodeling [47,48,49]. In a recent study, immunocytochemical techniques showed that carbohydrate epitopes are associated with the major cell wall polysaccharides and glycoproteins in the digestive glands of *Aldrovanda vesiculosa* and *Dionaea muscipula* [50,51]; however, up to now there is no detailed study about the immunocytochemistry of cell walls in mucilage emergences of carnivorous plants. 

In this study, we used *Drosophyllum lusitanicum* as a model to explore the immunocytochemistry of cell walls is mucilage glands.

## 2. Results

### 2.1. Structure of Stalked Mucilage Glands and Occurrence of Cell Wall Ingrowths 

**Figure 2:**
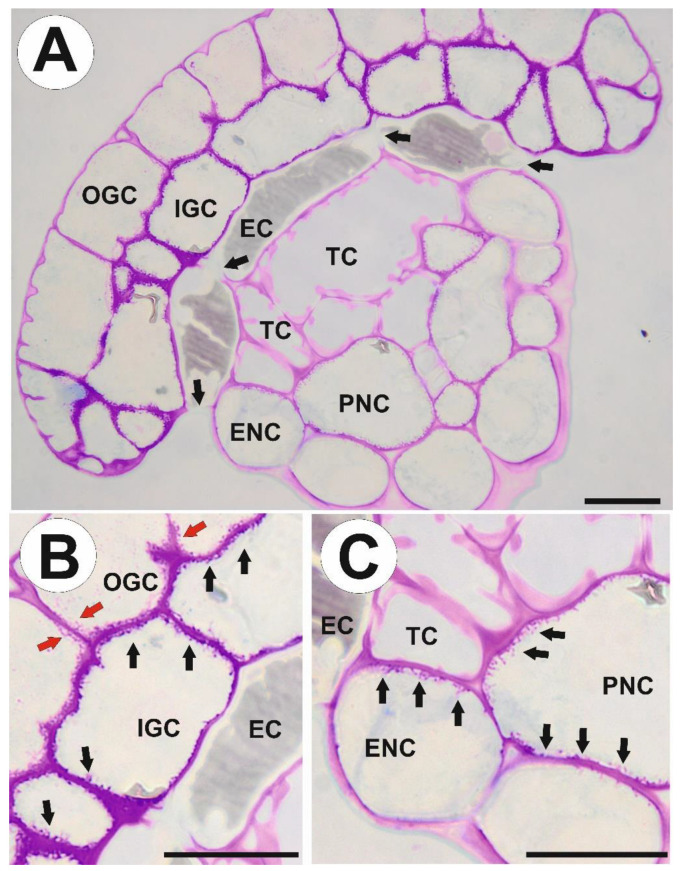
Gland anatomy and occurrence of cell wall ingrowths in mucilage gland cells of *Drosophyllum lusitanicum*. (**A**) Anatomy of glands; outer glandular cell (OGC), inner glandular cell (IGC), endodermoid cells (EC), Casparian strip (black arrows), tracheid (TC), epidermal neck cell (ENC), parenchymal neck cells (PNC), scale bar 20 µm. (**B**) Positive result of the PAS reaction of the cell wall ingrowths (red arrows) in outer glandular cell (OGC) and cell wall ingrowths (black arrows) in inner glandular cell (IGC), endodermoid cells (EC), scale bar 20 µm. (**C**) Positive result of the PAS reaction of the cell wall ingrowths (black arrows) in epidermal neck cell (ENC), parenchymal neck cells (PNC), endodermoid cells (EC), tracheid (TC), scale bar 20 µm.

The mucilage gland (emergence) consists of a glandular head, neck zone (a connecting zone), and stalk (Figure 1C and Figure 2A). The gland head is formed by an outer and inner layer of glandular (secretory) cells and supported by a layer of endodermoid (barrier) cells. The endodermoid cells (which are modified with cutin endodermoid parts in anticlinal cell walls, similar to Casparian strips) have contact with a core of spongy tracheids with spiral-shaped thickenings. Lateral tracheids are surrounded by epidermal and parenchymal neck cells. In the stalk, xylem (the number of xylem cells depends on the size of the gland) and phloem are surrounded by two rows of cells, an external layer of larger epidermal cells and an inner layer of smaller parenchymal cells.

We used the periodic acid-Schiff (PAS) reaction to visualize cell wall ingrowths (Figure 2B,C). In outer glandular cells, cell wall ingrowths occur on the inner portion of the anticlinal cell walls and the inner periclinal wall. In inner glandular cells, cell wall ingrowths occur along the entire inner cell wall surface (Figure 2B). In epidermal neck cells, cell wall ingrowths occur on cell walls that have contact with tracheids and parenchymal neck cells (Figure 2C). In parenchymal neck cells, cell wall ingrowths occur along the entire inner cell wall surface (Figure 2C).

### 2.2. Distribution of Arabinogalactan Proteins (AGPs) in Mucilage Glands

**Figure 3:**
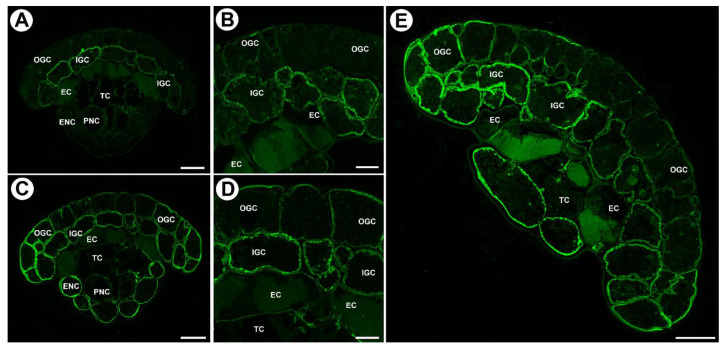
Arabinogalactan proteins detected in mucilage glands of *Drosophyllum lusitanicum*. (**A**,**B**) Arabinogalactan proteins (labeled with JIM14) were detected in the gland: outer glandular cell (OGC), inner glandular cell (IGC), endodermoid cells (EC), Casparian strip (black arrows), tracheid (TC), epidermal neck cell (ENC), parenchymal neck cells (PNC), scale bars 20 and 10 µm, respectively. (**C**,**D**) Arabinogalactan proteins (labeled with JIM8) were detected in the gland: outer glandular cell (OGC), inner glandular cell (IGC), endodermoid cells (EC), tracheid (TC), epidermal neck cell (ENC), parenchymal neck cells (PNC), scale bars 20 and 10 µm, respectively. (**E**) Arabinogalactan proteins (labeled with JIM13) were detected in the gland: outer glandular cell (OGC), inner glandular cell (IGC), endodermoid cells (EC), tracheid (TC), epidermal neck cell (ENC), parenchymal neck cells (PNC), scale bar 20 µm.

We used JIM8, JIM13, and JIM14 antibodies in order to localize AGPs (Table 1). JIM14 yields strong fluorescence signals of AGP epitopes in the walls of inner glandular cells, especially in the wall ingrowths (Figure 3A,B). In the walls of outer glandular cells, the fluorescence signal was much weaker (Figure 3A,B). There was a lack of signal in endodermoid cells. In tracheids, AGP epitopes recognized by JIM14 occurred in primary cell walls but not in secondary cell walls (Figure 3A). The delicate fluorescence signal of this AGP also occurred in the cell walls of neck epidermal cells and parenchymal cells. 

JIM8, in contrast, gave a strong fluorescence signal of AGP epitopes in the walls of both outer and inner glandular cells (Figure 3C,D): the signal occurred in cell wall ribs and wall ingrowths (Figure 3C,D). In addition, the AGP signal occurred in the walls of endodermoid cells except for modified, endodermoid parts of cell walls. There was a lack of signal in tracheids. AGP was observed in cell wall ingrowths in neck epidermal cells and parenchymal cells. 

The AGP epitope that JIM13 recognizes was present in the cell walls of both outer and inner glandular cells. It occurred in cell wall ingrowths and also cell wall ribs (Figure 3E). In addition, this AGP was found in the walls of endodermoid cells except for modified endodermoid parts of cell walls. It was absent in tracheids. AGP occurred in cell wall ingrowths in neck epidermal cells and parenchymal cells (Figure 3E).

**Figure 4:**
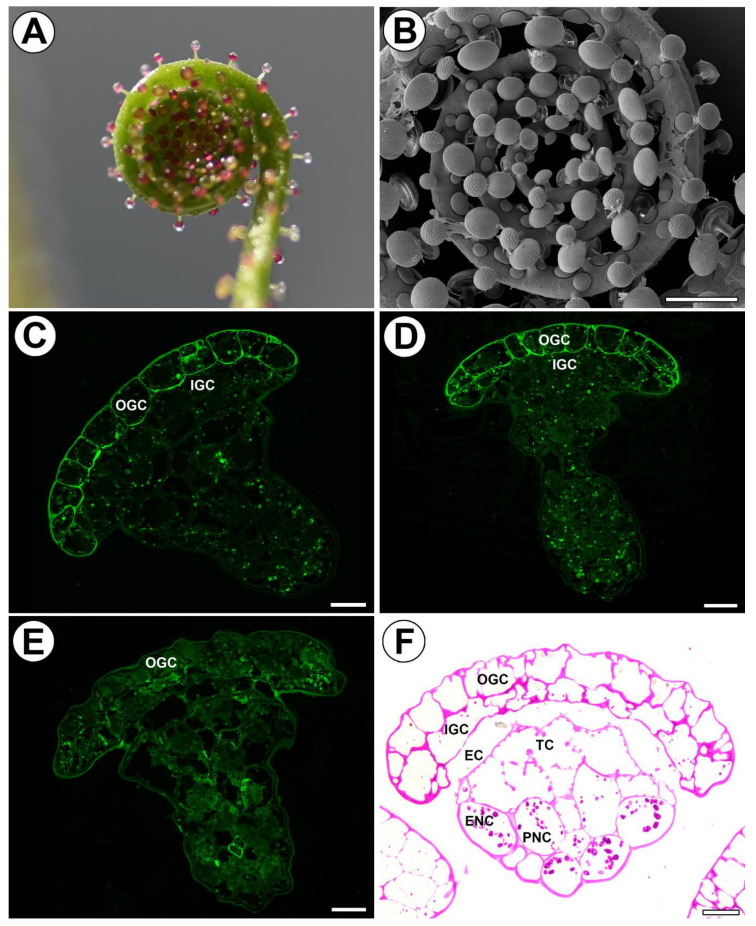
Arabinogalactan proteins detected in young mucilage glands of *Drosophyllum lusitanicum*. (**A**) Twisted top of a young leaf. (**B**) Twisted top of a young leaf seen in SEM, scale bar 1 mm. (**C**) JIM8 labeled arabinogalactan proteins in the following cells of the young gland: outer glandular cell (OGC), inner glandular cell (IGC), scale bar 20 µm. (**D**) JIM13 labeled arabinogalactan proteins in the outer glandular cell (OGC), inner glandular cell (IGC), scale bar 20 µm. (**E**) JIM14 labeled arabinogalactan proteins in outer glandular cell (OGC), scale bar 20 µm. (**F**) PAS reaction in the head of a young gland. The wall ingrowths had not formed yet, outer glandular cell (OGC), inner glandular cell (IGC), endodermoid cells (EC), tracheid (TC), epidermal neck cell (ENC), parenchymal neck cells (PNC), scale bar 20 µm.

In young glands (from the rolled-up part of the leaf, Figure 4A,B) AGPs recognized by JIM8 and JIM13 occurred in cell walls of outer glandular cells and in cytoplasmic content in various cell types of the gland (Figure 4C,D). AGPs recognized by JIM14 occurred mainly in cytoplasmic content in various cell types of the gland (Figure 4E). In cells of these glands, cell wall ingrowths had not formed yet (Figure 4F).

### 2.3. Distribution of Homogalacturonan in Mucilage Glands

**Figure 5:**
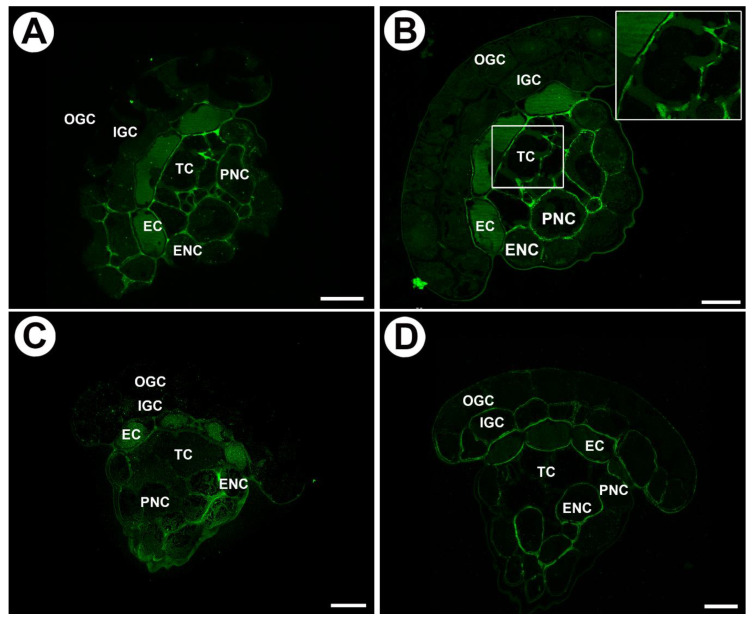
Homogalacturonan (HG) detected in mucilage glands of *Drosophyllum lusitanicum*. (**A**) HG (labeled with JIM5) was detected in a gland: outer glandular cell (OGC), inner glandular cell (IGC), endodermoid cells (EC), tracheid (TC), epidermal neck cell (ENC), parenchymal neck cells (PNC), scale bar 20 µm. (**B**) HG (labeled with LM19) detected in a gland: outer glandular cell (OGC), inner glandular cell (IGC), endodermoid cells (EC), tracheid (TC), epidermal neck cell (ENC), parenchymal neck cells (PNC), scale bar 20 µm. (**C**) HG (labeled with JIM7) detected in a gland: outer glandular cell (OGC), inner glandular cell (IGC), endodermoid cells (EC), tracheid (TC), epidermal neck cell (ENC), parenchymal neck cells (PNC), scale bar 20 µm. (**D**) HG (labeled with LM5) detected in a gland: outer glandular cell (OGC), inner glandular cell (IGC), endodermoid cells (EC), tracheid (TC), epidermal neck cell (ENC), parenchymal neck cells (PNC), scale bar 20 µm.

Low methyl esterified homogalacturonans (HG) were detected by JIM5 and JIM19 antibodies (Table 1). JIM5 reacted with strong fluorescence in the cell walls of tracheids except for secondary cell walls (Figure 5A). In addition, epitopes were detected in cell walls of endodermal cells and neck parenchyma cells (Figure 5A). There was weak fluorescence in the cell walls of some inner glandular cells, whereas outer glandular cells showed no reaction. The delicate fluorescence signal detected by LM19 (low methyl esterified HGs) occurred in the external cell walls of outer and inner glandular cells (Figure 5B). 

In a similar way, JIM19 gave a strong signal in the cell walls of tracheids (except for secondary cell wall thickenings) and also in neck parenchyma cells. A signal occurred in the walls of endodermoid cells except for modified, endodermoid parts of cell walls. In addition, a strong fluorescence signal occurred in cell wall ingrowths in neck parenchyma cells, and in some neck epidermal cells (Figure 5B). 

Highly esterified HGs were detected by JIM7 antibodies. An intense fluorescence signal was observed in the cell walls of endodermoid cells, neck, epidermal, and parenchyma cells (Figure 5C). The external cell walls of inner glandular cells gave only a weak signal. The pectic polysaccharide (1–4)-β-D-galactan was detected by LM5. It yielded significant fluorescence signals in the cell walls of the endodermoid cells (except for modified, endodermoid parts of cell walls) and neck parenchyma cells (Figure 5D), in addition to cell walls of internal glandular cells. A weak signal was detected in the cell walls of neck epidermal cells and external cell walls of outer glandular cells (Figure 5D).

### 2.4. Distribution of Hemicellulose in Mucilage Glands

**Figure 6:**
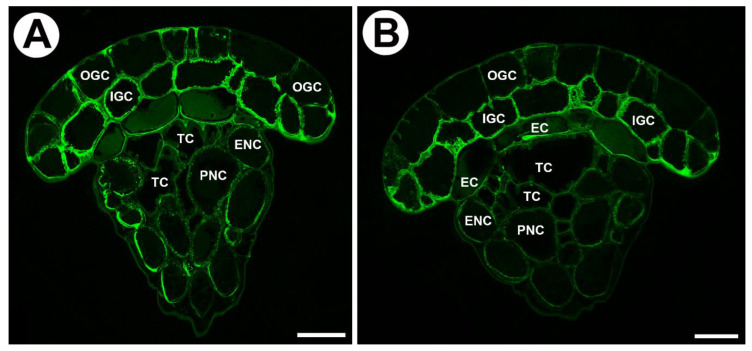
Xyloglucan detected in mucilage glands of *Drosophyllum lusitanicum*. (**A**) Xyloglucan (labeled with LM15) detected in a gland: outer glandular cell (OGC), inner glandular cell (IGC), endodermoid cells (EC), tracheid (TC), epidermal neck cell (ENC), parenchymal neck cells (PNC), scale bar 20 µm. (**B**) Xyloglucan (labeled with LM25) detected in a gland: outer glandular cell (OGC), inner glandular cell (IGC), endodermoid cells (EC), tracheid (TC), epidermal neck cell (ENC), parenchymal neck cells (PNC), scale bar 20 µm.

Xyloglucan was detected by LM15 and LM25 antibodies (Table 1). LM15 gave a strong fluorescence signal in the walls of the inner glandular cells (Figure 6A), especially in wall ingrowths. Also in outer glandular cells, this epitope occurred in cell walls, cell wall ribs, and cell wall ingrowths (Figure 6A). In neck epidermal and parenchymal cells, the cell wall ingrowths reacted positively. It occurred in the primary cell walls of tracheids as well as in the walls of endodermoid cells except for modified, endodermoid parts of cell walls (Figure 6A).

LM25 gave a strong fluorescence signal from xyloglucan in the walls of the inner glandular cells (Figure 6B), especially in the wall ingrowths. Also, the wall ingrowths of neck epidermal and parenchymal cells stained brightly (Figure 6B). Glandular cells, cell walls, and cell wall ribs were stained (Figure 6B). A weak signal also occurred in the walls of endodermoid cells except for modified, endodermoid parts of cell walls, and in primary cell walls of tracheids. 

### 2.5. Enzymatic Treatments

**Figure 7:**
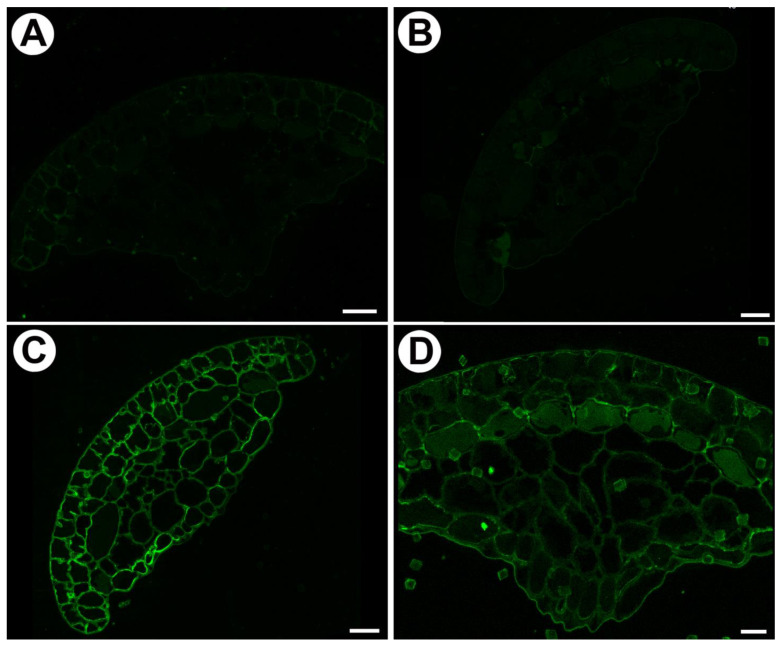
Homogalacturonan and xyloglucan detected in the mucilage glands of the *Drosophyllum lusitanicum* after pre-treatment with pectate lyase. (**A**) HG detection (labeled with JIM5), scale bar 20 µm. (**B**) HG detection (labeled with JIM7), scale bar 20 µm. (**C**) Xyloglucan detection (labeled with LM15), scale bar 20 µm. (**D**) Xyloglucan detection (labeled with LM25), scale bar 20 µm.

When sections were pre-treated with pectate lyase (pectins were removed) no signals from JIM5 and JIM7 were observed (Figure 7A,B). Occurrence of xyloglucan, which was detected by LM15 and LM25 antibodies, was similar in no treated (Figure 6A,B) and pre-treated with pectate lyase sections (Figure 7A,B).

## 3. Discussion

### 3.1. Cell Wall Polymers and Gland Cell Types

Heslop-Harrison [32] proposed a schematic structural model of the secretory gland of a carnivorous plant. Such a gland consists of the following components: 1. secretory cells; 2. endodermis (cell, or layer of cells, each with a Casparian strip); 3. reservoir (cell or tissue); 4. vascular supply. Each of these elements has a different structure and function. According to Juniper et al. [1], in the stalked glands of *Drosophyllum* and *Drosera,* the tracheids in the gland head are equivalent to reservoir cells. Juniper et al. [1] also highlighted differences in the ultrastructure of individual cell types of carnivorous plant glands. Therefore, it is interesting to determine whether individual gland components (cell types) have their own wall polymer patterns. Here, we investigated mucilage producing glands of *Drosophyllum lusitanicum* and detected differences in the wall components of different cell types. Generally, cell walls of secretory cells of *Drosophyllum* are weak in methylesterified and de-esterified HG pectins, which is in contrast to cell walls of endodermoid cells, tracheids, and neck cells. Also, in *Aldrovanda* digestive glands, the cell walls of secretory head cells were poor in HG epitopes recognized by JIM5 and JIM7 [51]. Similar observations were made in stellate trichomes of *Dionaea* [52] and bifid trichomes of *Aldrovanda* [53]. In addition, in *Drosophyllum* gland tracheids there are differences in the occurrence of epitopes between the primary cell wall and the secondary (lignified) cell wall, which is seen in the occurrence of pectins (detected by JIM19 and JIM5) and xyloglucan (detected by LM15). It is uncertain whether there is a lack of epitopes in the wall or whether lignification blocks the availability of specific wall components to antibodies. Cell walls of secretory cells of *Drosophyllum* are enriched with arabinogalactan proteins, as in various glands of *Dionaea* [51,52] and *Aldrovanda* [50,54]. This may be related to the high activity of these cells (exocytosis), but also to the presence of cell wall ingrowths. Especially in *Aldrovanda* digestive glands, AGPs were abundant in different cell types, which had in common that they were transfer cells [50]. However, it should be noted that in *Drosophyllum* there is a difference between the occurrence of AGPs between outer and inner secretory cells. Especially inner glandular cells are rich in AGPs. This is probably connected with better-developed cell wall ingrowths in inner glandular cells. Also, the pectic polysaccharide (1–4)-β-D-galactan (detected by LM5) has a specific distribution and is observed mainly in the cell walls of the endodermoid cells, neck parenchyma cells, and internal glandular cells.

### 3.2. Cell Wall Polymers and Cell Wall Ingrowths

In plants, symplasmic transport of substances between cells is promoted by cell wall ingrowths and an amplified surface area of the plasma membrane. Accordingly, such cells, which are characterized by an intricate wall labyrinth (cell wall ingrowths and an amplified surface area of plasma membrane, and numerous mitochondria), are called transfer cells; they can support high symplasmic transport between plant cells [34,54]. Transfer cells are common characteristics of glands of carnivorous plants. Moreover, it is known that secretory cells of *Drosophyllum* glands are transfer cells [1]. Here, we established that not only secretory cells but also epidermal and parenchymal neck cells may be regarded as transfer cells because of their wall ingrowths. Concerning the molecular composition of the wall ingrowths, there is known to be variability in occurrence, abundance, and types of polymers among both angiosperms and bryophytes [50,51,55,56,57,58,59]; however, the state of knowledge is still not sufficient. We observed that the cell wall ingrowths in the transfer cells of *Drosophyllum* mucilage glands are rich in AGPs. The wall ingrowths were similar in their AGP composition (JIM8, JIM13, JIM14) to the wall ingrowths in the transfer cells of other carnivorous plants such as *Dionaea muscipula* [51] and *Aldrovanda vesiculosa* [50] as well as other plant species [55,57,60]. Our work demonstrated that the hemicelluloses recognized by the LM25 antibodies (for galactoxyloglucan) and LM15 antibodies (for xyloglucan) were present in the cell wall ingrowths in cells of *Drosophyllum* mucilage glands, which is similar to transfer cells of *Aldrovanda* [50]. These xyloglucans were also recorded in the cell wall ingrowths in transfer cells of bryophytes [57,58,59]. We found that homogalacturonan is absent in the cell wall ingrowths in *Drosophyllum* mucilage gland cells, similar to *Aldrovanda* transfer cells [50]. However, homogalacturonan was recorded in cell wall ingrowths in transfer cells of *Vicia faba* [55], *Pisum sativum* [60], and bryophytes [57,58,59]. Reported observations on cell wall architecture in different groups of plants and our studies on carnivorous plants show that the cell walls (including cell wall ingrowths) are highly dynamic and structurally heterogeneous structures. Further studies on a larger group of species will determine whether the presence or absence of certain components in cell walls has a physiological and ecological rationale or may reflect the relatedness or evolutionary position of a particular group of plants. So far, it has been established that AGPs are prevalent in the cell wall ingrowths in various unrelated groups of plants.

### 3.3. Cell Wall Polymers and Gland Activity

Cell wall polymers are dynamic components and can be restructured during plant development and aging, e.g., [61,62,63,64,65,66,67], but also during stresses and plant interactions with other organisms [68,69,70,71,72,73]. Therefore, cell wall modifications must be taken into consideration in carnivorous plants, in which it has been proposed that the carnivory syndrome evolved via the evolution of an ancient defense pathway (and thus a stress response), because some carnivorous plants use defense-related processes to form an active digestive system; hence, cell death was replaced by prey digestion and nutrient acquisition [74,75,76].

Sticky fly-paper traps need to produce mucilage in their glandular cells. A highly active endomembrane system with Golgi bodies produces slime-containing vesicles. These vesicles are secreted by exocytosis through the plasma membrane, whose area is largely increased by the labyrinthine cell wall ingrowths. Also, the apoplast of the glands is important; in *Drosera*, it was suggested that the wall ingrowths serve as reservoirs for secreted substances [1,77,78]. During trap functioning, modifications of the labyrinthine cell walls were observed in *Dionaea* [79] and *Utricularia* [80]. Thus, specific plant cell wall molecules probably also have an essential role in all these changes. Because mucilage is transported via exocytosis, there should also be membrane recycling; thus, AGPs should occur. In gland cells of *Drosera capensis*, Samaj [81] detected AGPs using JIM13 antibodies and proposed that they might be involved in vesicle trafficking and membrane recycling. Olmos et al. [82] proved that AGPs play a role in vesicle trafficking. According to Wang et al. [83], AGPs act as extracellular cargo receptors and cross the plasma membrane to initiate endocytosis. We found accumulations of AGPs in glandular cells in *Drosophyllum* glands, but also in *Dionaea muscipula* [51] and *Aldrovanda vesiculosa* glands [50]. In *Dionaea muscipula,* there were differences in the occurrence of AGPs (labeled with JIM8 and JIM13) in the walls of gland secretory cells between unfed and fed traps [51]. It should be underlined that important changes also occur in digestive glands in traps of carnivorous plants. The cytomorphological changes (called ‘aggregation’) in the gland cells of the traps can be observed after prey is captured using light microscopy, and have been noted by several authors, e.g., Darwin [7] and Åkerman [84]. Traditional ultrastructural studies, e.g., [1,46,77,78,85], but also more recent studies based on samples cryo-fixed by high-pressure freezing [86,87,88,89], revealed that secretory cells of carnivorous plant glands have high activity, and there are changes of their endomembrane system that occur after trap activation and prey digestion.

Cytomorphological changes occur especially during gland development and maturation [52,77], particularly in mucilage glands that produce mucilage before trap activation. In both *Drosera* and *Drosophyllum* gland cells, the changes in the Golgi apparatus occur before and during mucilage production [42,77,90,91,92,93]. We found in *Drosophyllum* that AGPs occur in glandular cells in both mature and juvenile mucilage glands, but the pattern of AGP occurrence was different: in juvenile mucilage glands, AGPs occurred mainly in the cytoplasm of outer glandular cells. In these cells, wall ingrowths have not yet developed. This is in accordance with the report of Juniper et al. [1], who stated that labyrinthine walls (cell wall ingrowths) are laid down very late in carnivorous plant gland development. In mature glands of *Drosophyllum*, AGPs seem to accumulate in the cell wall ingrowths. We therefore suggest that the occurrence of AGPs in the cytoplasm of young cells (observed at the light microscopy level) may be connected with the later formation of cell wall ingrowths and that AGPs play a role in forming cell wall ingrowths. This is in accordance with the suggestion of McCurdy that AGPs participate in coordinating the required localized assembly of wall components [94].

## 4. Materials and Methods 

### 4.1. Plant Material

*Drosophyllum lusitanicum* (L.) Link plants were grown in greenhouses of the Botanical Garden of the Jagiellonian University. Plants were kept under high sunlight exposure.

### 4.2. Histological and Immunochemical Analysis

Leaf fragments (from three plants) were fixed in 8% (*w*/*v*) paraformaldehyde (PFA, Sigma-Aldrich, Poznań, Poland) mixed with 0.25% (*v*/*v*) glutaraldehyde (GA, Sigma-Aldrich) in PIPES buffer overnight at 4 °C. The PIPES buffer contained 50 mM PIPES (piperazine-N,N′-bis (2-ethanesulfonic acid), Sigma-Aldrich), 10 mM EGTA (ethylene glycol-bis(β-aminoethyl ether)N,N,N′,N′-tetraacetic acid, Sigma Aldrich), and 1 mM MgCl_2_ (Sigma-Aldrich), pH 6.8. For analysis of the occurrence of the major cell wall polysaccharides and glycoproteins, the plant material was embedded in LR White Resin (Polysciences Europe GmbH, Hirschberg an der Bergstrasse, Germany); infiltration was repeated twice, followed by polymerization at 55 °C, and sectioning (0.7–0.9 µm thick). The rehydrated sections in PBS buffer were blocked with 1% bovine serum albumin (BSA, Sigma-Aldrich) in a PBS buffer and incubated with the following primary antibodies overnight at 4 °C: anti-AGP—JIM8, JIM13, JIM14 [95,96,97,98]; anti-pectin—JIM5, JIM7, LM19, LM5 [95,99,100,101]; and anti-hemicelluloses—LM25, LM15 [99,100,101]. All of the primary antibodies were used in a 1:20 dilution. They were purchased from Plant Probes, UK, and the goat anti-rat secondary antibody conjugated with FITC was purchased from Abcam (Cambridge, UK). The chromatin in the nuclei was stained with 7 µg/mL DAPI (Sigma-Aldrich) diluted in a PBS buffer. The samples were then cover-slipped using a Mowiol mounting medium: a mixture of Mowiol ^®^4-88 (Sigma-Aldrich) and glycerol for fluorescence microscopy (Merck, Warsaw, Poland) with the addition of 2.5% DABCO (Carl Roth GmbH + Co. KG, Karlsruhe, Germany). They were viewed using a Leica STELLARIS 5 WLL confocal microscope with lightning deconvolution. At least two different replications were performed for each of the analyzed traps, and about five to ten sections from each organ were analyzed for each antibody used. Negative controls were created by omitting the primary antibody step, which caused no fluorescence signal in any of the control frames for any stained slides (Figure S1). To remove HG from cell walls, sections were pretreated with 0.1 M sodium carbonate pH = 11.4 for 2 h at room temperature. This was followed by digestion with a pectate lyase 10A (Nzytech) at 10 μg/mL for 2 h at room temperature in 50 mM N-cyclohexyl-3-aminopropane sulfonic acid (CAPS) with addition of 2 mM CaCl_2_ buffer at pH 10 [99] and then incubated with JIM5, JIM7, LM15, and LM25 antibody as described above.

### 4.3. Light Microscopy

Semi-thin sections (0.9–1.0 µm thick) were prepared for light microscopy (LM) and stained for general histology using aqueous methylene blue/azure II (MB/AII) for 1–2 min [102]. The periodic acid-Schiff (PAS) reaction was used to reveal the presence of any insoluble polysaccharides [103].

## 5. Conclusions

Here, we described in detail the differences in occurrence of cell wall components between various cell types in mucilage glands of *Drosophyllum lusitanicum.* Our cytological study showed that despite different gland functions, in the case of cell wall components, the glandular cells of *Drosophyllum lusitanicum* mucilage glands are similar to secretory cells of digestive glands of *Aldrovanda vesiculosa* and *Dionaea muscipula*. We established that the pattern of cell wall components is similar in glands of different carnivorous plants irrespective of their function (mucilage/digestive). Cell walls of secretory cells are poor in methylesterified and de-esterified pectins but enriched with hemicelluloses and arabinogalactan proteins.

## Figures and Tables

**Table 1:** Results of immunocytochemistry in the different cell types of the mucilage glands.

Cell Wall Polysaccharides and Glycoproteins	Gland Cell Types					
	Cell Walls of Outer Glandular Cell	Cell Walls of Inner Glandular Cell	Cell Walls of Endodermoid Cell	Cell Walls of Tracheid	Cell Walls of Epidermal Neck Cell	Cell Walls of Parenchymal Neck Cell
Arabinogalactan proteins	Occurred especially in cell wall ingrowths; in case of JIM14, weaker signal in cell walls	Occurred especially in cell wall ingrowths	Lack (JIM14); occurred (JIM8, JIM13)	Occurred (JIM14); lack (JIM8, JIM13)	Occurred especially in cell wall ingrowths (LM19)	Occurred especially in cell wall ingrowths (LM19)
Low methyl esterified homogalacturonans	Lack or weak signal	Lack or weak signal	Occurred	Occurred	Occurred	Occurred
Highly esterified homogalacturonans	Lack	Lack or weak signal	Occurred	Occurred	Occurred	Occurred
Galactan	Weak signal	Occurred	Occurred	Lack	Weak signal	Occurred
Xyloglucan	Occurred; strong signal in cell wall ingrowths	Occurred; strong signal in cell wall ingrowths	Occurred	Occurred	Occurred	Occurred

## Data Availability

Additional data is available from the correspondent author.

## Supplementary Materials

The following supporting information can be downloaded at: https://www.mdpi.com/article/10.3390/ijms242015045/s1.
