# Supplementary material for: Differences in the Occurrence of Cell Wall Components between Distinct Cell Types in Glands of Drosophyllum lusitanicum
Source: Int J Mol Sci. 2023 Oct 10;24(20):15045. doi: 10.3390/ijms242015045 (PMC10606540; doi:10.3390/ijms242015045)

Figure S1

Figure S1. Control reactions of cell wall components after immunolabeling. (**A,B**) Mucilage glands, bar 20 μm.

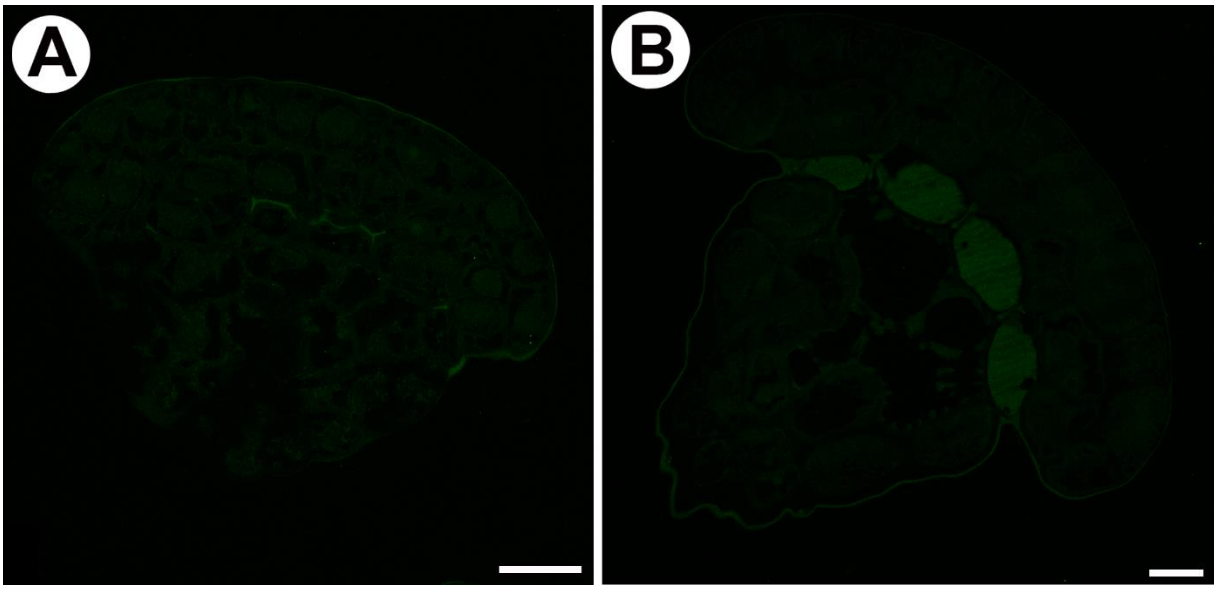

Supplement: Supplementary file 1 [file ijms-24-15045-s001.zip › ijms-2582231-supplementary.pdf]
